# Essay on Irregularity of the Teeth

**Published:** 1852-10

**Authors:** E. G. Tucker


### ARTICLE VI.

*Essay on Irregularity of the Teeth:* By E. G. TUCKER, M.D. Read before the American Society of Dental Surgeons, at the Thirteenth Annual Meeting, held at Newport, R. I., August, 1852.

MR. PRESIDENT AND GENTLEMEN:—

It was with a proper mistrust of my own ability to afford you instruction, that I consented to address you on any subject at the present time. I did not consent with any conviction, that I should render a service that might not be better given by others; but, rather from a sense of duty that the humblest member of society owes to himself and to those with whom he may be associated—always to use, at all times, his best endeavors to do what he can to promote a common object.

The American Dental Society was not, I presume to say, formed for the display of mere theoretical knowledge, but, rather, with a more utilitarian purpose, that of interchanging the practical results of a common experience, and of communicating opinions without ceremony and without a spirit of rivalry, in regard to the vital interests of our profession. I speak only for myself, as you are all better acquainted with the opinions of others than I am. It will not be expected that I should tax you with any fragments of learning, knowing full well where the whole is possessed, the parts are not wanting.

In speaking of *irregularity of teeth*, I claim your attention upon a subject which has an importance in itself, and which requires rather the application of an ordinary knowledge, than any great exertion of genius. In the spirit of a praiseworthy ambition to discover new things, we are apt, *very apt*, to hold old ones in contempt. In attempting to accomplish some end, for notoriety, we are too much given to the habit of neglecting what many might denominate *trifles*. It will be conceded, I think, that in making out a course of duty for himself, a dentist should avoid the unworthy motives which slight the items of common knowledge, or which prevent difficult attainments, because, in the language of Wall and State streets, *they do not pay*. Our profession, like all others, has its barren duties, as well as its profitable opportunities. We must take it as it is, doing everything required by science, and neglecting nothing which shall promote the refinements and comforts of humanity. The littles make up the creditable aggregate, and no item, however small, should be disregarded. It is a recent thing to attempt to *regulate the natural growth of the teeth*, as if they were to be made an exception to everything else. To remove a tooth, has been considered as equivalent to the loss of a *tooth*, as if members were regarded in the light of compensation for irregularity. The farmer thinks of his nursery and his vegetable bed, that a growth may not be stinted, and a crop destroyed. A

horticulturist cuts a surplus vine or bud, that the flower may thrive and put forth its full measure of beauty, or that the fruit may mature in its highest perfection. It is not to be expected, that the dentist can remodel the defective jaw, or give unquestioned beauty to badly formed teeth; but no one will deny, that he can very much improve what may be termed unfortunate formations. It is a difficulty which all must deplore, that parents generally deem the teeth simply instruments of use, but not of care. They are not prized until they begin to fail, or are entirely gone.

Irregular teeth are lamented, indeed, but few, however, ever seriously look for a remedy, which too many believe beyond the reach of human agency. Some err, in thinking that the teeth of a child may be made to grow in harmony, but give no thought to teeth of a maturer period. This is a great mistake. Of difficult cases within the sphere of my practice, five-tenths of the patients have been from sixteen to twenty-five years of age. Out of sixty cases, I have not lost more than three teeth, and these were affected previously by disease. We cannot always command desired results; but it is not reasonable to allow our failures to discourage all proper application of skill. If the *pulling rings*, which I have commended to the profession, do not invariably result in improvement, we have no right to infer that their use is to be condemned. Such a principle would lead us to discard all but literally sovereign remedies, known only to the credulous and the quack.

In the early part of my professional life, I was led to consider the best means of saving the teeth. That is generally deemed an act of friendship on the part of a dentist—ought to be considered a professional duty. People do not think of a doctor until they become diseased; and all are comparatively indifferent as to his opinions in respect to preventives. So of the dentist! His aid is not asked, except to remove and to remedy an evil which already exists. Having two difficult cases, some six years since, I was led particularly to study the best means to be employed in managing them. The spiral springs were too stiff, or too yielding, and the gold bands were difficult to adjust. I looked for a substance that would accommodate itself, if I may so express myself, to the circumstances of the case. A substance that would exert a steady power, and adapt itself to the variations of a changing growth in the teeth, which were the subject of treatment, I could think of nothing more proper than India-rubber; and with a view to obtain rings of various extent, I ordered *tubes* to be made of different sizes, which, by cutting transversly, I had, at once, a class of instruments—the simplest of the simple, and the cheapest of the cheap. They were easy of application; and, with improvements such as I have been able to make, they have proved to be *efficient* for all ordinary cases of irregularity.

In favoring the use of India-rubber, tubes or rings, I would not be understood as underrating all other contrivances, or any of them. I speak an honest opinion, founded on my own experience. Let it be received for what it is worth. I ask no commendation that contributes to personal consideration, or the motives which are not common to the profession. Though simple, these rings cannot be used with advantage, unless applied with the utmost care. Indeed, a careless application of them may create new difficulties, or aggravate old ones. The exact position of the teeth, the line of force to be observed, and the tensity of power to be exerted, are all considerations requiring study—and a careful judgment. I submit the means for your consideration and trial, well knowing you are too practical in your views to decide upon their usefulness without a knowledge, which, alone, can come from experience and observation, and that you will not be inclined, hastily, to condemn any method of practice which promises a common good, without a patient and unprejudiced examination.

It may seem to a superficial person as favoring too much the profession, if we were to recommend both to the parent and physician, an early resort to the dentist for advice and service, in reference to the teeth of children, and, yet, such a course would result in economy and comfort. There would be saving of money, and a security against pain. A child should be early placed in the care of the dentist, that he may be spared the premature removal of the teeth, on account of a troublesome pain, and that no means necessary for the preservation of the teeth should be neglected. We are, necessarily, compelled to guard against two extremes—an unreasonable solicitude, on the one hand, and culpable neglect on the other. Mothers are apt to look for immediate remedies, where time, alone, is necessary, with common prudence; or, to suppose, that because their own teeth required no regulating, the same is true of their children.

A slight soreness or inflammation of the gums may often be relieved by some astringent, until the proper period arrives for the removal of the offending member. As a general rule, no tooth should be extracted until its successor is near at hand. In my humble opinion, there is one rule which should always be observed, viz. To extract no temporary tooth from a child unless absolutely necessary for some special reason, such as ulceration, &c. This is done too frequently, and I feel persuaded that it is the duty of the dentist to condemn the practice. The progress of science depends much upon the exercise of an independent judgment. Dentistry is a profession requiring both science and mechanical skill. We are responsible for its instrumentality. We are not mere agents to answer the specific request of patients who require our services in a particular way, and who, at the same time, hold us responsible for any failure. If a dentist be truly fitted for his profession, he owes a duty to science, to use his best judgment in all cases; and, oftentimes, without regard to the preconceived opinions of those who are the subject of his care. Otherwise, he is constantly liable to annoyance of complaints, for consequences for which he is not answerable, and misrepresentations for which he cannot account; unless his knowledge entitles him to be chief judge in all cases, he might as well save time, by omitting all inquiries which are supposed to result in the surest means for meliorating the conditions of the patients. For no consideration, whatever, should the dentist be persuaded to please his patrons against his own deliberate judgment. Such a course may sometimes excite the conceited patient, who calls upon the dentist as a mere *me-*

*chanic;* but, generally, it would be approved, and ensure a safe practice, and tend to establish the respectability of the profession.

Comparatively speaking, dentistry is a new profession, and hence, the importance of opening new sources, which shall reach every variety of mind engaged in its practice. There may be individuals who have always kept by themselves, and who might have important suggestions to make, if we could but reach them. I would advise a wide survey for discovery, that no one may be neglected, who has genius and skill, and a disposition for inquiry. Such a line of conduct may lead, not only to the improvement of others, but result in profit to ourselves. I am no friend to professional jealousy—to that stinted view of things which commences with suspicion, and ends with contempt. It is unworthy of the votaries of science—unworthy of man, when considered in his moral relations, as a being responsible for his motives and his acts. We sometimes avoid men because of preconceived opinions which are unfavorable to their standing or capacity, when, perhaps, a more disinterested mood of intercourse might oftentimes bring within our sphere, persons eminently fitted for professional distinction, and who require no aid but the right hand of fellowship, to ensure a confidence, and to command a skill.

Genius is limited to no class—success confined to no family. The brightest gem is sometimes thrown up by the humblest miner; and it is not for us to say, who is to be, or, *who is not to be*, the leader in discovery—the greatest in our art, or the profoundest in his inquiries to advance science, and to elevate the profession with which we are proud to be identified.

